# Transcriptomics and Lipid Metabolomics Analysis of Subcutaneous, Visceral, and Abdominal Adipose Tissues of Beef Cattle

**DOI:** 10.3390/genes14010037

**Published:** 2022-12-22

**Authors:** Lili Du, Tianpeng Chang, Bingxing An, Mang Liang, Tianyu Deng, Keanning Li, Sheng Cao, Yueying Du, Xue Gao, Lingyang Xu, Lupei Zhang, Junya Li, Huijiang Gao

**Affiliations:** 1 Institute of Animal Sciences, Chinese Academy of Agricultural Sciences, Beijing 100193, China; 2 College of Computer and Information Engineering, Tianjin Agricultural University, Tianjin 300000, China; 3 College of Animal Science and Technology, Qingdao Agricultural University, Qingdao 266000, China

**Keywords:** transcriptomics, lipid metabolomics, adipose tissue, beef cattle

## Abstract

Fat deposition traits are influenced by genetics and environment, which affect meat quality, growth rate, and energy metabolism of domestic animals. However, at present, the molecular mechanism of fat deposition is not entirely understood in beef cattle. Therefore, the current study conducted transcriptomics and lipid metabolomics analysis of subcutaneous, visceral, and abdominal adipose tissue (SAT, VAT, and AAT) of Huaxi cattle to investigate the differences among these adipose tissues and systematically explore how candidate genes interact with metabolites to affect fat deposition. These results demonstrated that compared with SAT, the gene expression patterns and metabolite contents of VAT and AAT were more consistent. Particularly, *SCD* expression, monounsaturated fatty acid (MUFA) and triglyceride (TG) content were higher in SAT, whereas *PCK1* expression and the contents of saturated fatty acid (SFA), diacylglycerol (DG), and lysoglycerophosphocholine (LPC) were higher in VAT. Notably, in contrast to *PCK1*, 10 candidates including *SCD*, *ELOVL6*, *ACACA*, and *FABP7* were identified to affect fat deposition through positively regulating MUFA and TG, and negatively regulating SFA, DG, and LPC. These findings uncovered novel gene resources and offered a theoretical basis for future investigation of fat deposition in beef cattle.

## 1. Introduction

Adipose tissue, known as a metabolically heterogeneous endocrine organ mostly composed of adipocytes [1], performs a crucial role in lipid metabolism, energy regulation, temperature maintenance, insulin resistance, and meat quality of farm animals [2,3]. In mammals, adipose tissue is temporal and tissue-specific [4,5]. Anatomically, adipose tissue could be classified subcutaneous adipose tissue (SAT), visceral adipose tissue (VAT), abdominal adipose tissue (AAT), intramuscular adipose tissue, intermuscular adipose tissue, heart adipose tissue, and kidney adipose tissue. These adipose tissues displayed cellular, molecular, physiological, and functional differences [6]. Notably, the larger proportion of small adipocytes in SAT resulted in its higher insulin sensitivity and a sharper absorption of circulating fatty acids (FAs) and triglycerides (TGs), which could prevent fat deposition in non-adipose tissue [7]. Studies reported higher concentrations of SAT contributed to being protective against metabolic diseases, conversely, VAT accumulation was prone to metabolic syndrome [8]. Additionally, SAT could protect the carcass from cold shortening and drip loss during cooling [9], whereas excessive abdominal fat was considered to be economic loss [10].

Currently, the RNA sequencing (RNA-seq) technique has become a powerful tool for quantitative analysis of the whole transcriptome expression profile, which is successfully applied to the complex traits of farm animals like fat deposition and fatty acid traits of beef cattle [11,12]. The differential expression level of lipogenic genes had been proven to have an effect on lipid metabolism in bovine adipocytes [13], such as *SCD*, *FABP4*, and *LPL* in Japanese Black and Holstein cattle [14]. As the ultimate omics level of biological systems, metabolomics has a distinct advantage in measuring the final products of genetic and environmental interactions, and then screens significant metabolic molecules as biomarkers to reflect the interrelation between the changes of biomarkers and phenotypic traits [15], which has been widely used in biomedicine [16], food nutrition [17], and crop trait selection, breeding, and evaluation [18,19]. Notably, a relevant report emphasized lipidomics was a secondary omics rather than a discipline separate from metabolomics as the units it studied were metabolites [20]. In comparison with other omics, lipidomics was still undergoing development as its relevant tools, databases, and informatics resources were not yet perfect. Previous work showed lipidomics had been applied to elucidate the lipid profile changes in brown and white adipose tissue [21,22]. Additionally, several studies reported the marker metabolites of intramuscular fat in beef cattle, such as capric acid and 3-phosphoglyceric acid in Japanese black cattle [23], oxysterol in Angus, Hereford and Wagyu × Angus [24], and glucose, choline, and acetyl groups in wagyu cross steers [25]. However, there were no reports on the metabolomics of SAT, VAT, and AAT in beef cattle.

**Figure 1:**
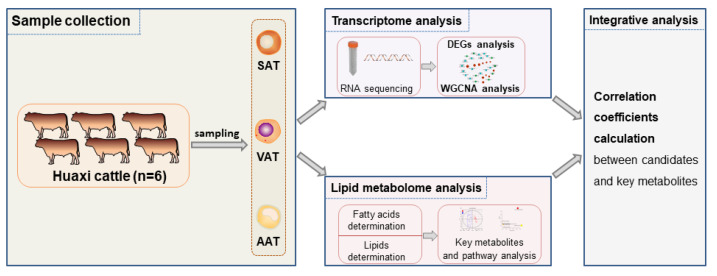
Technology roadmap. SAT: subcutaneous adipose tissue; VAT: visceral adipose tissue; AAT: abdominal adipose tissue; DEGs: differentially expressed genes; WGCNA: weighted gene co-expression network.

With the development of single omics technology, multi-omics research has become a hotspot to systematically explore the genetic regulatory mechanism of the quantitative traits of livestock, which could improve the comprehensibility and reliability of the results and facilitate the progress of animal breeding. The integrated transcriptomics and metabolomics research on grass-fed and grain-fed Angus cattle reported that the meat produced by grass-fed beef was more tender and had a higher omega3/omega6 ratio than grain-fed beef [26]. Another transcriptomic and metabolomic study of adipose tissue in goats showed that L-carnitine could promote the differentiation and thermogenesis of cells in brown adipose tissue via activating the AMPK signaling pathway while reducing lipid accumulation by inducing lipolysis and thermogenesis of cells in white adipose tissue [27]. Here, this study collected three types of adipose tissues (SAT, VAT, and AAT) to identify candidate genes and key metabolites for fat deposition in beef cattle via a comprehensive analysis of transcriptomic and lipid metabolomics (Figure 1), which not only offered a data basis for further understanding the characteristics and differences of different adipose tissues of beef cattle, but provided novel insights for the gene mapping strategy in other livestock.

## 2. Materials and Methods

### 2.1. Ethics Statement

All the experimental procedures relating to animals were authorized by the Ethics Committee of the Institute of Animal Sciences, Chinese Academy of Agricultural Sciences (IAS, CAAS), Beijing, China (approval number: IAS2020-48).

### 2.2. Animals and Sampling

The 73 experimental individuals slaughtered in this study were all from Huaxi cattle resource population established in Inner Mongolia Aokesi Livestock Breeding Co., Ltd. (Inner Mongolia, China) After weaning, the cattle were raised under the same fattening strategies (Feed composition: corn silage, corn straw, wine tank, soybean meal, corn tablet, concentrated feed, and concentrate supplement) and unified standardized management according to GB/T 27642–2011, and then slaughtered in Zhongao Food Co., Ltd. (Aohan Banner, Chifeng, China). The SAT, VAT, and AAT of six healthy bulls with an average age of 26 months and an average weight of 700 kg were immediately collected and frozen in liquid nitrogen for the extraction of total RNA, fatty acid, and lipid, respectively.

### 2.3. Transcriptomic Analysis

The raw sequencing reads underwent quality assessment using FastQC (v0.11.9) and then Trimmomatic (v0.39) was performed to filter reads containing ploy-N, adaptors sequences, and low-quality reads to gain clean reads. Using Hisat2 (v2.2.1) to anchor clean reads to the reference genome of *Bos taurus* (BTA) ARS-UCD1.2 (ftp://ftp.ensembl.org/pub/release-101/fasta/bos_taurus/DNA/ (accessed on 6 January 2022)) [28]. SAMtools (v1.11) was applied for converting SAM files to BAM files. The read counts were quantified with FeatureCounts (v1.5.2) [29]. Differentially expressed genes (DEGs) in different adipose tissue groups (SAT-VAT, SAT-AAT, and VAT-AAT) were identified by DESeq2 (v1.18.0) package [30]. Benjamini-Hochberg’s approach for multiple hypothesis testing was applied to adjust the *p*-value (*p-adj*). Genes with adjusted *p*-value (*p-adj*) < 0.05 and |log2FoldChange| ≥ 1 were recognized as DEGs. Notably, the unannotated DEGs were discarded after mapping to the BTA reference genome. Furthermore, the gene expression level was quantified by fragments per kilobase of transcript per million mapped reads (FPKM). All non-redundant DEGs were selected to construct gene co-expression networks via the weighted gene co-expression network (WGCNA) package [31]. Here, the relevant parameters were set as previously described [32]: (1) the soft threshold (*β*) was selected to construct the scale-free network when the fit index (R^2^) reached 0.8; (2) the minimum number of genes per module was 30; (3) the threshold value for similar module merging was set to 0.25.

### 2.4. Functional Enrichment Analysis

ClusterProfiler (v4.0.0) package was used to implement Gene Ontology (GO) and Kyoto Encyclopedia of Genes and Genomes (KEGG) enrichment analyses of DEGs and significant modules obtained by WGCNA, respectively [33]. GO terms and pathways with *p* < 0.05 and q < 0.05 were considered to be significantly enriched. Particularly, the PPAR signaling pathway and biological processes of lipid metabolism were worthy of great concern [34]. The protein–protein interaction (PPI) network of candidate genes was constructed via the STRING database (https://string-db.org/ (accessed on 4 February 2022)) to explore the interaction relationship between DEGs.

### 2.5. Metabolite Content Determination

#### 2.5.1. Fatty Acid Species and Content Determination

The fatty acid in each sample was extracted in the Central Laboratory Platform of the IAS of CAAS following their standard procedures. The fatty acid content (*X*) was expressed as grams per 100 g (g/100 g) and its calculation formula was as follows:

$$X_{i}=\left(\frac{A_{i}\times C_{si}}{A_{si}}\right)\times \frac{f\times F_{FAME-FA}}{m/1\times d}\times 0.001=\frac{C_{i}\times f\times F_{FAME-FA}}{m/1\times d}\times 0.001$$

$X_{i}$: the content of each fatty acid in each sample; $A_{i}$: the peak area of each fatty acid methyl ester in each sample; $C_{si}$: the concentration of fatty acid methyl ester standard in the standard determination fluid in milligrams per kilogram (mg/kg); $C_{i}$: the concentration of each fatty acid methyl ester in the sample liquid calculated from the standard curve, in mg/kg; $A_{si}$—the peak area of each fatty acid methyl ester standard contained in the standard determination fluid; f: the proportion of each fatty acid in the total standard fluid; $F_{FAME-FA}$: the conversion coefficient of each fatty acid methyl ester to fatty acid; d: the internal standard factor; 0.0001: the coefficient of fatty acid content per gram converted to fatty acid content per 100 g of sample.

#### 2.5.2. Lipid Species and Content Determination

The lipid components in SAT, VAT, and AAT were extracted by a three-phase (MTBE-methanol-water) solvent system combined with tissue homogenization technology. Lipids were detected using ultra-high performance liquid chromatography-high resolution mass spectrometry (UPLC-HRMS) via ultimate TM 3000 ultra-performance liquid chromatography-tandem quadrupole-electrostatic field orbital trap high-resolution mass spectrometry (Thermo Scientific, Waltham, MA, USA). An Accucore C30 core-shell column was utilized for lipid molecules’ chromatographic separation under 50 °C which were eluted with 60% acetonitrile in water (A) and 10% acetonitrile in isopropanol (B) both containing 10 mM ammonium formate and 0.1% formate. Positive and negative ion electrospray ionization was used to detect mass spectrometry (MS). The same amount of lipid extract from each sample was taken to be mixed and centrifuged to prepare the pooled quality control (QC) sample for data quality control in the untargeted lipidomics testing. In this study, four QC samples were evenly inserted into the analysis sequence. The sample extract, lipid identification, and quantification analyses were conducted at Beijing BioMiao Biotechnology Co., Ltd. (Beijing, China).

### 2.6. Lipidomics Raw Data Preprocessing 

LipidSearch software (v4.1) was conducted to process untargeted lipidomics data including peak picking and lipid identification. The detected MS2 spectrum was searched against in silico predicted spectra of a diverse phospholipid, neutral glycerolipid, sphingolipid, glycosphingolipids, steroids, etc. The mass accuracy for precursor and MS/MS product ions searching were set at 5 ppm and 5 mDa, respectively. TraceFinder software (v4.1; Thermo Scientific, Waltham, MA, USA) was used to extract the area under curve (AUC) of all lipid molecules for lipid quantitative, and then the AUC data were transferred to Excel software (Microsoft, Redmond, WA, USA) for data normalization using a linear regression algorithm.

### 2.7. Lipid Metabolomic Profiling Analysis

The lipidomic data deriving from different measurements were merged and those detected with multiple methods were excluded to ensure the uniqueness of lipid, and then Log2 was transformed for final statistical analysis. SIMCA (v14.1; Umetrics, Umea, Sweden) and OmicShare tools (https://www.omicshare.com/tools/ (accessed on 24 February 2022)) were employed to process orthogonal partial least squares discriminant analysis (OPLS-DA) on three adipose tissues. The differently accumulated lipids (DALs) were selected with variable importance in projection (VIP) > 1 and the independent sample *t*-test *p*-value < 0.05 [35]. Lastly, using MetaboAnalyst (https://www.metaboanalyst.ca (accessed on 26 February 2022)) for lipidomic pathway analysis of DALs [36].

### 2.8. Transcriptomic and Lipid Metabolomic Integration Analysis

The Spearman correlation coefficients (SPCC) were calculated for the integrative analysis between the FPKM expression of candidate genes and the content of metabolites including important fatty acids and DALs. Gene-metabolite correlation with the criteria |SPCC| > 0.5 and *p* < 0.05 will be selected for further investigation.

## 3. Results

### 3.1. The Evaluation of Sequencing Data

In the present study, 14 samples (five SAT, four VAT, and five AAT) were finally selected for transcriptome analysis according to the sequencing quality assessment of adipose tissue. After quality control (Table S1), 93.91G clean sequence was obtained. The average values of Q20 and Q30 were 97.81% and 94.00%, respectively, and the average GC content was 52.19%. The alignment of clean data to the BTA reference genome (ARS-UCD1.2) showed that the total mapping rate of each sample was over 93%, further indicating the quality of clean data was high and could be used for transcriptome analysis.

### 3.2. Principal Component Analysis among Three Groups

Principal component analysis was conducted on the gene expression level of samples in SAT-VAT (Figure S1a), SAT-AAT (Figure S1b), and VAT-AAT (Figure S1c) groups. In the three groups, the variation of gene expression explained by PC1 and PC2 was more than 50% and 15%, respectively, illustrating the samples were grouped reasonably.

### 3.3. Identification of Differentially Expressed Genes among Three Groups

**Figure 2:**
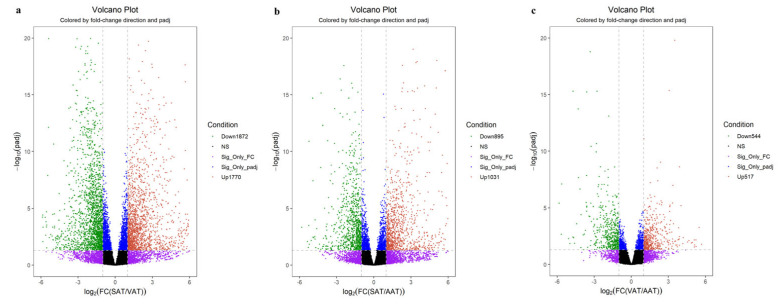
Volcano plots of differentially expressed genes in SAT−VAT (**a**), SAT−AAT (**b**), and VAT−AAT (**c**) comparative groups. The red and green dots represent the up-regulated and down-regulated genes, respectively.

**Figure 3:**
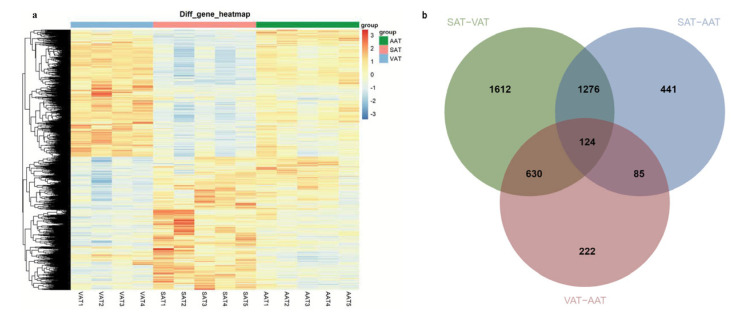
Heatmap (**a**) and Venn diagram (**b**) of differentially expressed genes.

With the classical criteria *p-adj* < 0.05 and |log_2_FoldChange| ≥ 1, a total of 3642, 1926, and 1061 DEGs were screened, respectively, in SAT-VAT (Figure 2a and Table S2), SAT-AAT (Figure 2b and Table S3), and VAT-AAT (Figure 2c and Table S4) groups. Hierarchical clustering analysis was performed on 4370 DEGs identified from the three groups (Figure 3a). The result showed the expression patterns of genes were similar within the three groups. Notably, compared with SAT, the gene expression patterns of VAT and AAT were more consistent. Additionally, 124 overlapped DEGs were identified in the three groups, including 119 coding genes, three lncRNA, and two pseudogenes, among which 110 annotated coding DEGs located in bovine autosomes were mainly focused on (Figure 3b and Table S5).

### 3.4. Functional Enrichment Analysis of Differentially Expressed Genes

**Figure 4:**
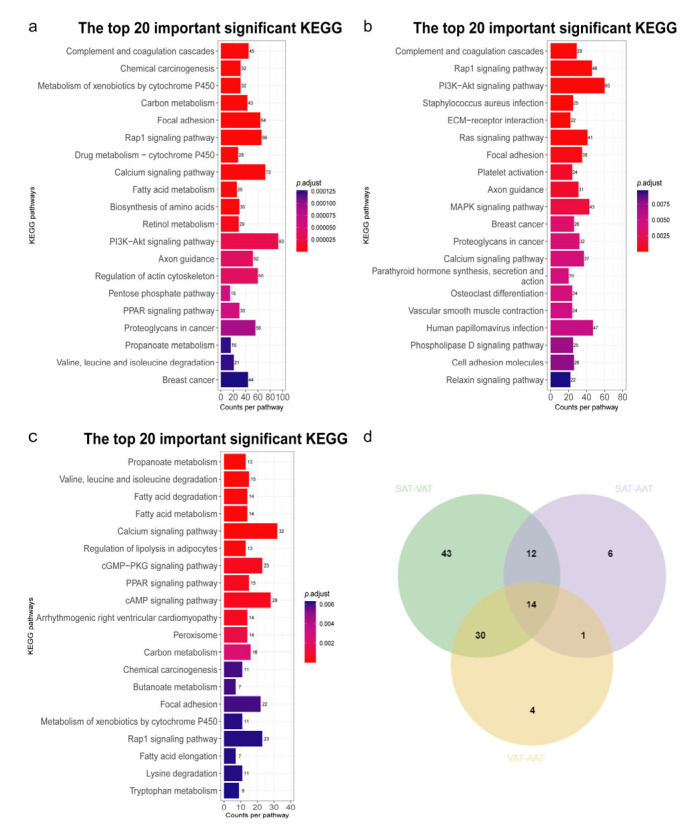
KEGG pathway enrichment of differentially expressed genes. (**a**–**d**) represent the enrichment and intersection of the KEGG pathway enriched by SAT-VAT, SAT-AAT, and VAT-AAT, respectively.

GO annotation performed on the DEGs identified in the SAT-VAT, SAT-AAT, and VAT-AAT groups demonstrated these DEGs were significantly enriched in 194, 133, and 20 GO terms, respectively (Table S6). The DEGs screened in the SAT-VAT group were associated with the organic acid metabolic process (GO:0006082), oxoacid metabolic process (GO:0043436), and carboxylic acid metabolic process (GO:0019752). For the SAT-AAT group, the DEGs were mainly involved in vasculature and blood vessel development (GO:0001944, GO:0001568), wound healing (GO:0042060), and cardiovascular system development (GO:0072358). Cell components and biological processes such as cell adhesion, movement, and migration were primarily enriched by the DEGs identified in the VAT-AAT group. Additionally, Figure 4a–c visualized the top 20 significant pathways enriched by these DEGs screened in the three groups. The details of pathway enrichment were listed in Table S7. A total of 14 overlapped pathways were enriched in the three groups (Figure 4d and Table 1). Notably, the PPAR signaling pathway played an important role in fat deposition. Therefore, in the three groups, three overlapped DEGs (*PCK1*, *PLIN4,* and *FABP7*) enriched in this pathway should be focused on.

### 3.5. Weighted Gene Co-Expression Network Analysis

#### 3.5.1. Adipose Tissue-Module Relationship Analysis

**Figure 5:**
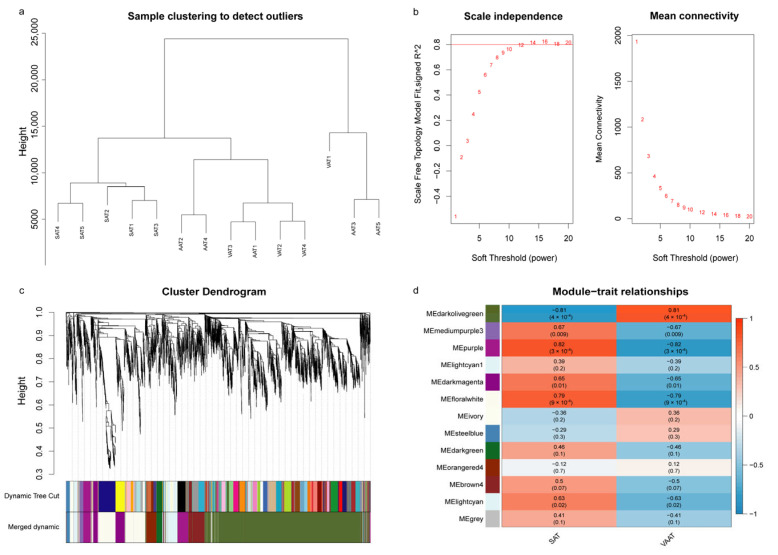
Weighted gene co-expression network analysis. (**a**) Sample cluster diagram; (**b**) Soft threshold calculation; (**c**) Functional module division; (**d**) Relationship between gene modules and adipose tissues. The value outside the parentheses expresses Pearson’s correlation coefficients between the module eigengenes and adipose tissues, and the number within the parentheses is the *p*-value.

The hierarchical clustering on the FPKM expression levels of 4370 DEGs identified in three groups showed SAT clustered separately, whereas VAT and AAT clustered more mixed, further implying that VAT and AAT had more similar structures or functions (Figure 5a). Therefore, VAT and AAT were merged into one group and renamed “VAAT” in this study. The “pickSoftThreshold” function was then used to calculate the *β* value. Finally, an appropriate value, *β* = 14, was determined for the scale-free network construction (Figure 5b). The dynamic-cutting method was used for functional module division, and a total of 12 functional modules were obtained (Figure 5c), six of which were significant modules, including darkolivegreen (|*r*| = 0.81, *p* = 4 × 10^−4^), mediumpurple3 (|*r*| = 0.67, *p* = 0.009), purple (|*r*| = 0.82, *p* = 3 × 10^-4^), darkmagenta (|*r*| = 0.65, *p* = 0.01), floralwhite (|*r*| = 0.79, *p* = 9 × 10^-4^), and lightcyan (|*r*| = 0.63, *p* = 0.02) (Figure 5d and Table S8). Except for the darkolivegreen module that had a strong negative correlation with SAT, the other five modules were significantly positively correlated with SAT and negatively correlated with VAAT. Of note, the genes in the grey module did not belong to any module.

#### 3.5.2. Significant Module Genes Analysis

**Figure 6:**
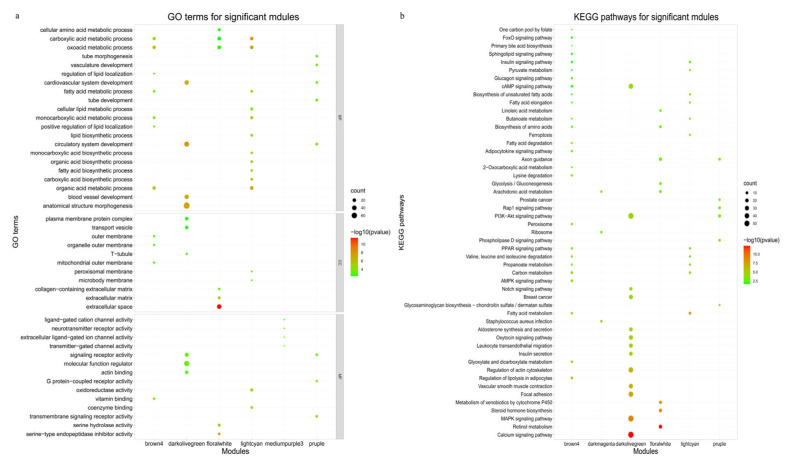
GO annotation (**a**) and KEGG pathway (**b**) of genes enriched in significant modules.

Functional enrichment analysis for the six significant module genes was firstly conducted with the “clusterProfile” package (Table S9). As illustrated in Figure 6a,b, genes in the lightcyan module were mainly involved in biological processes related to fat metabolism, including the fatty acid metabolic process (GO:0006631), organic acid metabolic process (GO:0006082), PPAR signaling pathway (bta03320), fatty acid metabolism (bta01212), etc. There were no significant GO terms and pathways enriched by the darkmagenta and medumpurple3 module genes, respectively. Genes in the darkolivegreen, purpl, and floralwhite modules were mainly enriched to pathways associated with signal transduction and disease occurrence. Taken together, the module functional enrichment analysis showed that the genes in the lightcyan module were mainly related to fat deposition; thus, this module was selected for the subsequent analysis. Table S10 listed the significantly enriched GO terms and pathways regarding fat deposition and their involved genes in the lightcyan module. Deleting duplicated genes, a total of 33 DEGs were determined in this module. Furthermore, according to the filter criteria with |gene significance (GS)| > 0.4 and |module membership (MM)| > 0.8, 90 hub genes were screened in this module. Combined with the above two conditions, a total of 17 genes such as *SCD*, *ELOVL6*, *ACACA*, *SLC27A6*, *ADIPOQ*, *OXCT1*, *PCK2*, *ACADSB,* and *EHHADH* were selected in the lightcyan module (Table 2). Among them, *RGN* and *PDHA1* would not be considered as key candidate genes affecting fat deposition as they were located on the sex chromosome. Therefore, 15 genes were initially screened as important candidates for further analysis.

### 3.6. Identification of Candidate Genes Affecting Fat Deposition

**Figure 7:**
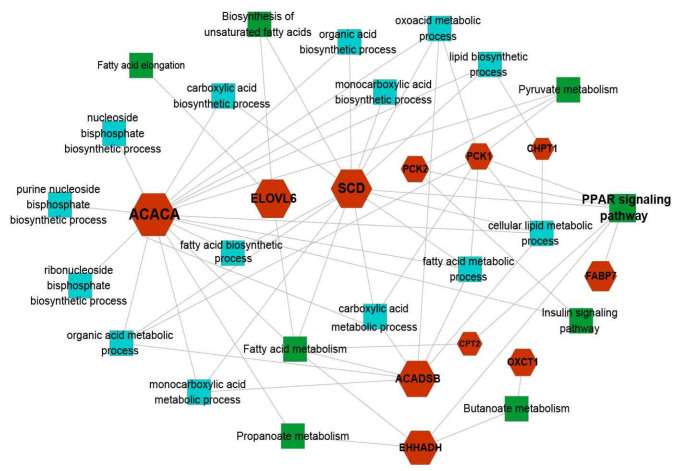
Eleven candidates involved in GO terms and KEGG pathways regarding fat deposition. The cyan and green squares, respectively, show GO terms and KEGG pathways. Genes marked in red represent candidate genes identified in this study.

Combined with DEGs and WGCNA analysis strategies among three adipose tissues, 18 potential candidate genes were screened to affect fat deposition, and their average expression levels in three adipose tissues were analyzed (Table S11). The results showed that *SCD* was abundantly expressed in SAT, whereas *PCK1* expression level was the highest in AAT. *TDH* and *SHC4* could be approximated as non-expressed genes as their expression levels in the three tissues were less than or close to 1. Therefore, through gene expression, literature investigation, and gene function, 15 genes including *SCD*, *ELOVL6*, *ACACA*, *OXCT1*, *ACADSB*, *EHHADH*, *FABP7*, *PLIN4*, *PCK1*, *PCK2*, *MBOAT7*, *PM20D1*, *CPT2*, *CHPT1*, and *PRKAR2A* were selected to construct PPI network for further determining the pivotal candidate genes (Figure S2). The thicker the interaction line, the higher the interaction score between the two genes. Those with confidence greater than 0.4 were regarded as moderately important interaction networks, and those with confidence greater than 0.9 were defined as important interaction networks [37]. There was a stronger interaction between *ACACA* and *ELOVL6* as the interaction confidence was as high as 0.838 (Table 3). *PLIN4*, *MBOAT7*, *PM20D1*, and *PRKAR2A* had no interaction with other genes. Therefore, 11 genes including *SCD*, *ELOVL6*, *ACACA*, *OXCT1*, *ACADSB*, *EHHADH*, *FABP7*, *PCK1*, *PCK2*, *CPT2*, and *CHPT1* were finally filtrated as candidates affecting fat deposition (Figure 7).

### 3.7. Metabolites Determination

#### 3.7.1. Fatty Acid Composition and Content

**Figure 8:**
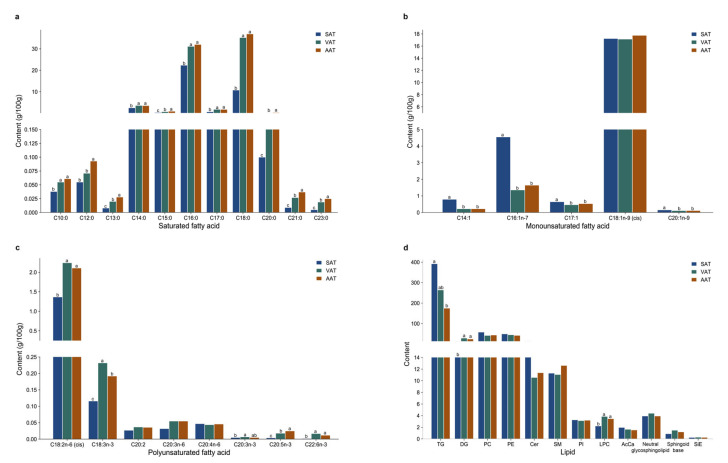
Fatty acid and lipid determination. (**a**–**c**) The composition and content of saturated fatty acid, monounsaturated fatty acid, and polyunsaturated fatty acid; (**d**) The composition and content of lipid. Different letters above the column represent significant differences in the content of metabolites (fatty acid or lipid) in different adipose tissues (*p* < 0.05), whereas the same letters indicate no significant differences in the content of metabolites (fatty acid or lipid) in different adipose tissues (*p* > 0.05).

In this study, twenty-four fatty acid species were detected in all three adipose tissues (Table S12), including 11, 5, and 8 kinds of saturated fatty acids (SFA), monounsaturated fatty acids (MUFA), and polyunsaturated fatty acids (PUFA), respectively, which correspondingly accounted for 59.49–76.91%, 20.34–37.94%, and 2.52–2.79% of the total fatty acid content. The fatty acid content analysis represented that there was no significant difference in SFA content between VAT and AAT; however, both of them were significantly higher than those in SAT (Figure 8a–c). Among them, palmitic acid (C16:0) and stearic acid (C18:0) were the two most important SFA constitutions. Conversely, the MUFA content in SAT was significantly higher than that in VAT, but there was no significant difference with that in AAT. Oleic acid (C18:1n-9) made up the highest proportion of MUFA, followed by palmitoleic acid (C16:1). The PUFA contents in the three adipose tissues were stable, all within 3% of the total content. The desaturation index (DI) of SAT was significantly higher than that of VAT and AAT, while the elongation index (EI) was opposite, which might be related to the lipogenic gene activity of adipose tissue.

#### 3.7.2. Lipid Composition and Content

Untargeted lipidomics technology was used to detect lipids from SAT, VAT, and AAT in the positive and negative ion modes. A total of 418 lipid species classified into 12 lipid classes were detected, including triglyceride (TG), glycerophosphocholine (PC), glycerophosphoethanolamine (PE), diacylglycerol (DG), ceramide (Cer), sphingomyelin (SM), glycerophosphoinositol (PI), lysoglycerophosphocholine (LPC), neutral glycosphingolipid, acyl carnitine (AcCA), sphingoid base, and sitosterol ester (SiE), among which TG was the main component of lipids making up 50.48% (Figure S3). QC was used to standardize the lipid data. As shown in Figure 8d and Table S13, the TG content in SAT was significantly higher than those of VAT and AAT, while the relative contents of DG and LPC were significantly lower than those of VAT and AAT. There was no significant difference in the contents of 12 lipids classes between VAT and AAT, implying VAT and AAT were more similar.

### 3.8. Lipid Data Analysis

#### 3.8.1. Assessment of Lipid Data Quality

Plotting the time series diagram of the first principal component score of the QC sample. The small variation of PC1 of samples from QC1 to QC4 meant that the analysis quality of the platform was stable (Figure S4a). The relative standard deviation (RSD) of more than 94.0% of lipids was less than 30% (Figure S4b), further proving the quality of lipid data was excellent. Generally, R2 and Q2 should be higher than 0.5, and the difference between them should be less than 0.3. The R2 and Q2 in SAT-VAT and SAT-AAT groups demonstrated that the model had good interpretation and prediction ability (Figure S5a,b). However, for the VAT-AAT group (Figure S5c), the R2 and Q2 of the model represented that the difference between the VAT and AAT was smaller than that of the other two groups.

#### 3.8.2. Screening of Differentially Expressed Lipids among Three Groups

**Figure 9:**
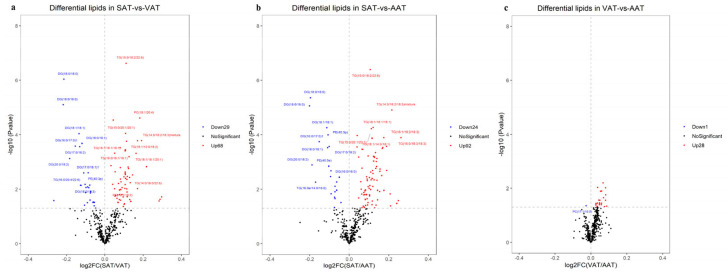
Volcano plots of differently accumulated lipids in SAT−VAT (**a**), SAT−AAT (**b**), and VAT−AAT (**c**) comparative groups.

Using the criteria with VIP > 1 and *p* < 0.05 (*t*-test) to screen DALs (Table S14). In SAT-VAT (Figure 9a), SAT-AAT (Figure 9b), and VAT-AAT (Figure 9c) groups, 97, 116, and 29 DALs, respectively, were identified. TG accounted for the highest proportion of the DALs. Additionally, two common DALs were identified among the three groups, which were TG (49:3) (monolinolenic triglyceride) and TG (54:5) (monostearic triglyceride). Therefore, TG played a decisive role in the development of fat deposition.

#### 3.8.3. Pathway Enrichment of Differentially Expressed Lipids

**Figure 10:**
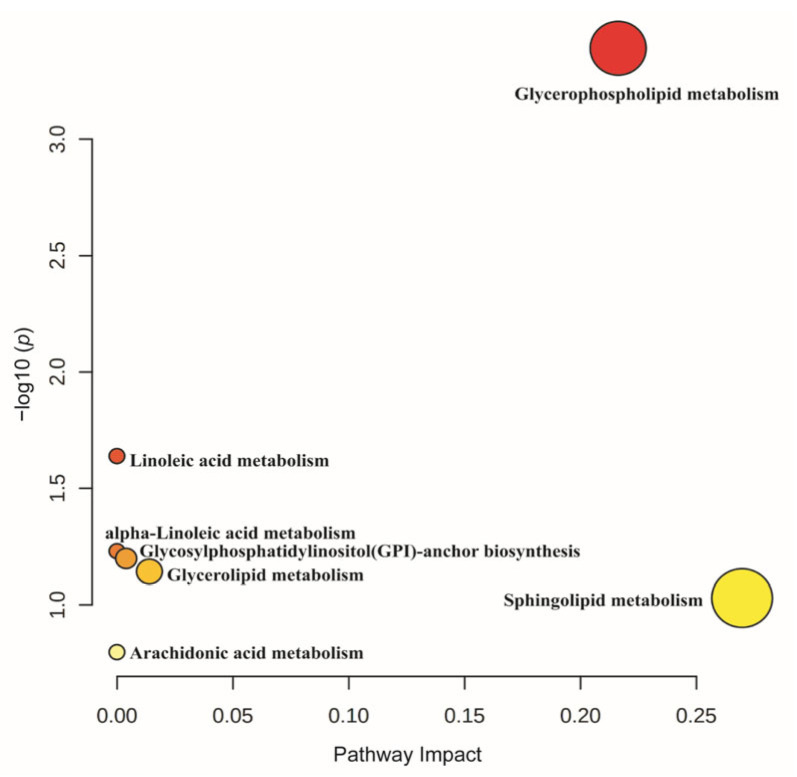
Pathway enrichment of all differently accumulated lipids identified by three groups.

MetaboAnalyst was used to perform pathway enrichment on the DALs identified in the three groups. A total of seven metabolic pathways were enriched (Figure 10), one of which was Glycosylphatidylinostiol (GPI)-anchor biosynthesis; three of which were enriched in pathways associated with lipid metabolism, including glycerophospholipid metabolism, glycerolipid metabolism, and sphingolipid metabolism; and three of which were enriched in pathways involved in unsaturated fatty acid metabolism, such as linoleic acid metabolism, α-Linoleic acid metabolism, and arachidonic acid metabolism. This study focused on pathways related to lipid metabolism and unsaturated fatty acid metabolism.

### 3.9. Transcriptomic and Lipid Metabolomic Integration Analysis

**Figure 11:**
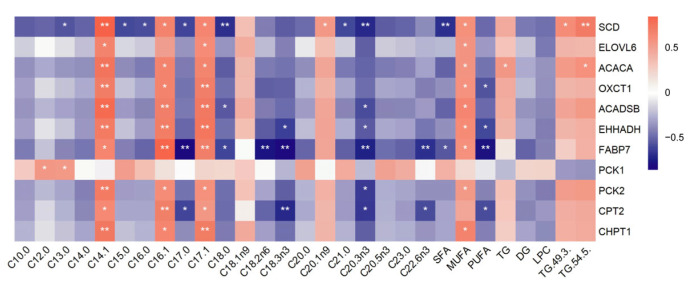
Correlation analysis between candidate genes related to fat deposition and differential fatty acids and lipids. The legend on the right shows the correlation coefficient. Red and blue represent the positive and negative correlation, respectively. The asterisk indicates significance: ** *p* < 0.01, * *p* < 0.05.

The Spearman correlation coefficients between 11 candidate genes and important metabolites including FAs and DALs were calculated for transcriptomic and lipid metabolomic integration analysis (Table S15). In contrast to *PCK1*, the other candidates had the same regulatory mode on FAs and DALs (Figure 11), namely, these genes had positive regulations with MUFA and TG, whereas negatively regulated SFA, PUFA, DG, and LPC. 

## 4. Discussion

**Figure 12:**
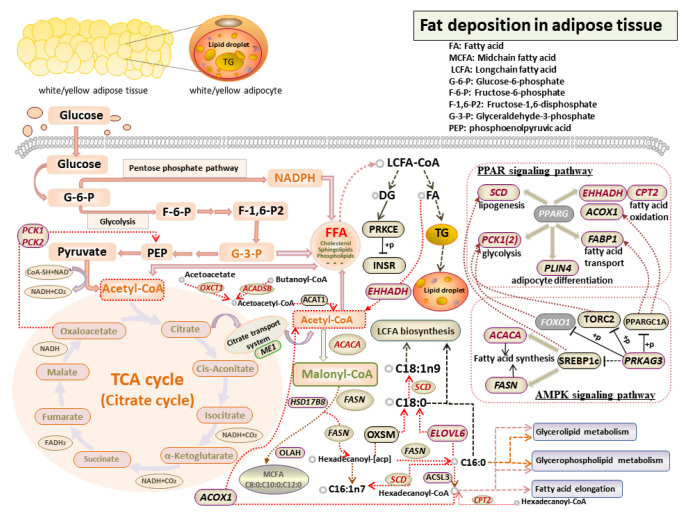
Map of the regulatory mechanisms of candidate genes regulating fat deposition.

Fat deposition occurs with the increase and differentiation of adipocytes, which was mediated by multiple adipogenic genes and catalytic enzymes [38,39]. Prior studies showed that the functional differences in different adipose tissues were caused by intrinsic gene expression [40]. The study of gene functions related to bovine fat deposition has become a prevalent research hotspot. In this study, through the transcriptomics and metabolomics analysis of SAT, VAT, and AAT, 11 candidate genes were identified to affect fat deposition via their involvement in fatty acid synthesis, metabolism and oxidation, pyruvate metabolism, and carbohydrate synthesis and digestion (Figure 12), including fatty acid synthesis genes (*ACACA*, *SCD*, and *ELOVL6*), fatty acid oxidation genes (*EHHADH* and *CPT2*), ketoacid metabolism genes (*ACADSB* and *OCXT1*), glycolytic genes (*PCK1* and *PCK2*), fatty acid transporter genes *FABP7*, and *CHPT1*. Additionally, it was further confirmed that PPAR signaling pathway was a pivotal pathway associated with fat deposition [41,42].

Fatty acid composition is a complex quantitative trait that determined the hardness and oiliness of adipose tissue and then affected the sensory quality and nutritional value of meat [43]. In mammalian cells, FAs undergo elongation, desaturation, *β*-oxidation, and peroxidation to synthesize complex lipids such as TG, phospholipids, and sphingolipids for mediating the stabilization of intracellular lipids [44]. Acetyl-CoA carboxylase (ACC) is a rate-limiting enzyme essential for de novo fatty acid synthesis. Its cytoplasmic isoform ACCα is encoded by acetyl-CoA carboxylase α (*ACACA*) gene [45]. The C-terminal carboxytransferase domain of *ACACA* catalyzed the carboxylation of acetyl-CoA to malonyl-CoA and, therefore, was involved in the first step of fatty acid synthesis [46]. This study presented that *ACACA* was positively correlated with total MUFA and TG, which roughly coincided with the study carried out by Zhang et al. (2010), who found that SNP3 genotype of *ACACA* was significantly correlated with TG in hybrid beef cattle [47]. Moreover, *ACACA* was significantly related to fat-related traits in beef cattle, such as backfat thickness [47] and intramuscular fat deposition [48]. Interestingly, in our previous studies, *ACACA* had been viewed as the most promising candidate affecting subcutaneous fat deposition in Chinese Simmental beef cattle [32]. In this study, *ACACA* was significantly enriched in multiple GO terms and pathways related to fat deposition and, thus, it was forecasted to be a molecular marker of fat deposition in beef cattle.

Two isoforms of stearoyl-CoA desaturase (*SCD*), namely *SCD1* and *SCD5*, have been identified in cattle, of which *SCD1* mRNA was highly expressed in adipose tissue [49]. Lehnert et al. (2007) reported that increased *SCD* activity in SAT would lead to high MUFA content [50]. Correspondingly, the current study showed that *SCD* was significantly positively related to MUFA content. Additionally, the C18:0 content in SAT was significantly lower than that of VAT and AAT, but the expression level of *SCD* was higher, which confirmed the increase of C18:0 inhibited *SCD* expression [51]. Therefore, the high expression of *SCD* in SAT was mainly caused by the lower SFA and higher MUFA contents in SAT. Previous studies had shown that *SCD* could regulate bovine FA content and TG synthesis [52,53]. *SCD* activity in bovine adipose tissue was positively correlated with the fatty acid desaturation index [54], which emerged as a regulator of the transformation from SFA to MUFA. MUFA is the major substrate for TG synthesis, hence it could be speculated that *SCD* exerted effects on fat deposition via regulating MUFA content.

Elongation of long-chain fatty acids family member 6 (*ELOVL6*) was another fatty acid synthesis gene identified in this study, which encoded an enzyme that was a key regulator of the first reaction in the elongation cycle of C12-16 long-chain fatty acids (LCFAs) in adipose tissue, such as catalyzing the extension of C16:0 to C18:0 and palmitoleic acid (C16:1n-7) to vaccenic acid (C18:1n-7) [55]. Interfering with *ELOVL6* increased the content of C16:0 but decreased the content of C18:1n9 [56]. Increased PUFA content significantly inhibited *ELOVL6* expression [57]. Consistent with these findings, this study demonstrated that *ELOVL6* had a positive correlation with the contents of a series of MUFA, such as C14:1, C16:1, C17:1, and C18:1n9, but negatively correlated with SFA and PUFA. Junjvlieke et al. (2020) reported that *ELOVL6* could regulate adipogenesis and lipolysis of bovine adipocytes through FoxO, cAMP, and Wnt signaling pathways [58]. It was found that the bovine *ELOVL6* gene activity was related to the expression of peroxisome proliferator activated receptor γ (PPARγ) in intramuscular fat [59] and subcutaneous fat [60]. In the study of Qinchuan cattle, *ELOVL6* activity was positively correlated with *PPARγ* expression, which promoted adipocyte proliferation in SAT and regulated lipid metabolism via regulating the expression of cell cycle genes [61]. Additionally, studies had also found that *ELOVL6* was not only an upstream inhibitor of lipid synthesis in perinatal dairy cows [62], but also a regulator of intramuscular fat deposition in yaks [63]. Consequently, *ELOVL6* could be recognized as a potentially important effector of fat deposition.

Fatty acid oxidation has also been recognized as the major biological process to regulate fat deposition. Intracellular fatty acid *β* oxidation begins with the regulation of the translocation of activated fatty acid acyl-CoA from the cytoplasm to the mitochondrial matrix, and this process was mediated by carnitine palmitoyltransferases 1 and 2 (CPT1 and CPT2) [64]. The expression of *CPT2* could be reduced when fatty acid oxidation stimulator *PPARγ* was silenced [65], indicating *CPT2* could participate in fatty acid oxidation by interacting with *PPARγ*. Enoyl-CoA hydratase and 3-hydroxyacyl CoA dehydrogenase (EHHADH) is essential for the production of medium-chain dicarboxylic acids. As a regulator, it was involved in the second and third steps of fatty acid *β*-oxidation [66]. Overexpression of *EHHADH* compensated for the functional defect of *HSD17B4*; therefore, it could replace *HSD17B4* to regulate the oxidation process of lauric acid (C12:0) [67]. *EHHADH* binding to *PPARα* provided a positive feedback loop to regulate the expression level of *PPARα* according to metabolic demands and then maintained lipid homeostasis [68]. 3-Oxoacid CoA-transferase 1 (OXCT1) is another rate-limiting enzyme indirectly involved in fatty acid *β*-oxidation through ketone body metabolism. In extrahepatic tissues, ketone bodies were decomposed by OXCT1 and eventually participated in the TCA cycle in the form of acetyl-CoA to produce ATP [69]. This process provided substrates for the biosynthesis of lipids and sterols in many tissues. Presently, research on *OXCT1* mainly focused on human cancers and ketoacidosis [69]; however, its functional study in adipose tissue of domestic animals was limited. Differential expression of *OXCT1* gene was identified in back adipose tissue of the F2 generation of Korean soil pigs and Yorkshire pigs [70]. Down-regulated *OXCT1* in ovine adipocytes promoted lipid accumulation, further emphasizing the importance of *OXCT1* in adipogenesis [71]. More interestingly, *OXCT1* expression was down-regulated in adipose tissue of obese subjects compared with healthy lean subjects [72]. Therefore, *OXCT1* might be involved in the TCA cycle through ketone body metabolism to affect fat deposition.

Acyl-coenzyme A dehydrogenase, short/branched-chain (ACADSB), found in mitochondria, regulates the catabolism of branched-chain amino acids and FAs [73]. *ACADSB* was used as a target for the diagnosis and treatment of malignant diseases. Down-regulation of *ACADSB* inhibited fatty acid catabolism and then led to lipid accumulation [74]. In bovine mammary epithelial cells (bMECs), *ACADSB* participated in the metabolism of TG and FA [75]. The contents of TG and FA in bMECs were reduced after the knockdown of this gene, demonstrating that *ACADSB* was a key factor affecting milk fat synthesis and lipid metabolism [76].

In vertebrates, two phosphoenolpyruvate carboxykinase (PEPCK) isoenzymes are detected, in which the cytosolic isoenzyme PEPCK-C and mitochondrial isoenzyme PEPCK-M are encoded, respectively, by phosphoenolpyruvate carboxykinase 1 (PCK1) and phosphoenolpyruvate carboxykinase 2 (PCK2) [77]. *PCK2* was recognized as a potential regulator of fat deposition in broilers [78]. PCK1 catalyzed oxaloacetate to phosphoenolpyruvate via GTP and then released GDP for energy metabolism [79]. This biological process could produce glycerol to regulate adipogenesis [80]. Additionally, *PCK1* was a target gene of *PPARγ* and regulated phosphoenolpyruvate through the PPARγ signaling pathway to affect the production of FAs [81], indicating *PCK1* could interact with *PPARγ* to participate in the processes concerning fat deposition [82]. Moreover, this gene had been screened to be associated with fat deposits in porcine [83] and chicken [37]. However, up to now, the regulatory mechanism of *PCK1* gene in bovine fat deposition was still unclear, and its function should be further explored.

Fatty acid binding proteins (FABPs) affect the uptake, transport, and metabolism of fatty acids. *FABP7* played an important role in the uptake of LCFAs in cell membranes [84]. Its high expression promoted cell proliferation by activating PPARγ signaling pathway [85]. *FABP3* and *FABP4*, members of the same family as *FABP7*, had been reported to be significantly associated with marbling, subcutaneous fat thickness, and fatty acid composition in cattle [86,87,88]. However, there were no studies reported that *FABP7* was related to fat deposition. In this study, *FABP7* was an overlapped DEG identified by transcriptome differential analysis between three groups, and it was significantly enriched in PPAR signaling pathway, which highlighted the importance of *FABP7* in fat deposition. Cholinephosphotransferase 1 (CHPT1) regulated choline metabolism by catalyzing phosphatidyl choline synthesis [89]. Inactivation of *CHPT1* could restrain phospholipid synthesis [90]; thus, this gene played a central role in bubble membrane formation, while its role in fat deposition was inconclusive.

## 5. Conclusions

Collectively, the integrative analysis of transcriptomics and lipid metabolomics in SAT, VAT, and AAT revealed 11 candidates including fatty acid synthesis genes (*ACACA*, *SCD*, and *ELOVL6*), fatty acid oxidation genes (*EHHADH* and *CPT2*), ketoacid metabolism genes (*ACADSB* and *OCXT1*), glycolytic genes (*PCK1* and *PCK2*), fatty acid transporter genes *FABP7*, and *CHPT1* were all identified to be involved in fatty acid synthesis, metabolism and oxidation, pyruvate metabolism, and carbohydrate synthesis and digestion, which directly or indirectly participated in TG synthesis and thus affected fat deposition. Among them, *FABP7* and *PCK1* were differentially expressed in three adipose tissues. These findings could provide deeper insights into the regulation mechanisms of fat deposition in beef cattle.

## Figures and Tables

**Table 1:** The overlapped significant pathways enriched by differentially expressed genes identified in SAT-VAT, SAT-AAT, and VAT-AAT groups.

ID	Description	Overlapped Genes ^1^
bta03320	PPAR signaling pathway	*PLIN4/FABP7/PCK1*
bta04911	Insulin secretion	*CACNA1C*
bta04010	MAPK signaling pathway	*KIT/CACNA1C/CACNA1H/RPS6KA6*
bta04151	PI3K-Akt signaling pathway	*PCK1/KIT/COMP/ITGB4*
bta04020	Calcium signaling pathway	*CACNA1C/CACNA1H*
bta04014	Ras signaling pathway	*KIT/PLA2G4B/PLD2*
bta04015	Rap1 signaling pathway	*KIT*
bta04512	ECM-receptor interaction	*COMP/ITGB4*
bta04510	Focal adhesion	*COMP/ITGB4*
bta04360	Axon guidance	*NGEF/NTNG2/BMP7/ROBO3*
bta05205	Proteoglycans in cancer	*TWIST1*
bta05224	Breast cancer	*KIT/HEY2*
bta05414	Dilated cardiomyopathy	*CACNA1C/ITGB4*
bta04934	Cushing syndrome	*CACNA1C/CACNA1H*

^1^ Overlapped genes referred to the DEGs identified in all three groups and enriched in this pathway.

**Table 2:** Hub genes associated with the fat deposit in the lightcyan module.

ID	Gene Symbol	BTA ^1^	GS ^2^	MM ^3^
ENSBTAG00000019625	*EHHADH*	1	0.59 (0.03)	0.96 (2.67 × 10^−8^)
ENSBTAG00000014649	*CPT2*	3	0.59 (0.03)	0.84 (1.91 × 10^−4^)
ENSBTAG00000002490	*CHPT1*	5	0.56 (0.04)	0.84 (1.77 × 10^−4^)
ENSBTAG00000010564	*ELOVL6*	6	0.52 (0.05)	0.98 (3.51 × 10^−10^)
ENSBTAG00000000011	*TDH*	8	0.57 (0.03)	0.91 (5.54 × 10^−6^)
ENSBTAG00000011934	*PCK2*	10	0.58 (0.03)	0.92 (3.90 × 10^−6^)
ENSBTAG00000022583	*SHC4*	10	0.74 (0.002)	0.89 (1.76 × 10^−5^)
ENSBTAG00000008734	*PM20D1*	16	0.69 (0.01)	0.92 (3.44 × 10^−6^)
ENSBTAG00000015908	*MBOAT7*	18	0.76 (0.002)	0.93 (1.67 × 10^−6^)
ENSBTAG00000017567	*ACACA*	19	0.51 (0.05)	0.96 (7.79 × 10^−8^)
ENSBTAG00000033186	*OXCT1*	20	0.56 (0.04)	0.99 (2.50 × 10^−11^)
ENSBTAG00000014205	*PRKAR2A*	22	0.53 (0.05)	0.88 (2.93 × 10^−5^)
ENSBTAG00000046753	*LOC107131843*	25	0.61 (0.02)	0.91 (5.93 × 10^−6^)
ENSBTAG00000055207	*SCD*	26	0.60 (0.02)	0.97 (9.99 × 10^−9^)
ENSBTAG00000018041	*ACADSB*	26	0.61 (0.02)	0.95 (1.74 × 10^−7^)
ENSBTAG00000046155	*RGN*	X	0.61 (0.02)	0.81 (4.20 × 10^−4^)
ENSBTAG00000019852	*PDHA1*	X	0.61 (0.02)	0.96 (5.01 × 10^−8^)

^1^ BTA, Bos taurus autosome; ^2^ GS, gene significance. The value outside the parentheses expresses the correlation coefficients between the gene expression and subcutaneous adipose tissue, and the number within the parentheses is the *p*-value; ^3^ MM, module membership. The value outside the parentheses expresses the correlation coefficients between the gene expression and module eigengene, and the number within the parentheses is the *p*-value.

**Table 3:** The confidence score among 11 candidate genes associated with fat deposition.

Node 1	Node 2	Confidence Score
*ACACA*	*ACADSB*	0.463
	*CPT2*	0.619
	*EHHADH*	0.608
	*ELOVL6*	0.838
	*PCK1*	0.543
	*PCK2*	0.407
	*SCD*	0.527
*ACADSB*	*CPT2*	0.670
	*EHHADH*	0.995
	*OXCT1*	0.426
*CHPT1*	*SCD*	0.523
*CPT2*	*EHHADH*	0.765
	*PCK1*	0.553
*EHHADH*	*ELOVL6*	0.486
	*FABP7*	0.533
	*OXCT1*	0.944
	*PCK1*	0.572
*PCK1*	*PCK2*	0.821

## Data Availability

The raw sequencing reads of subcutaneous, visceral, and abdominal adipose tissues have been submitted to Sequence Read Archive (SRA) with accession number PRJNA824614. Given that the paper has not yet been published, the data will be accessible on 1 May 2023, but can be obtained in advance from the corresponding author upon reasonable request.

## Supplementary Materials

The following supporting information can be downloaded at: https://www.mdpi.com/article/10.3390/genes14010037/s1, Figure S1 Principal component analyses of samples in SAT-VAT (a), SAT-AAT (b), and VAT-AAT (c) comparative groups; Figure S2 Protein-protein interaction network of 15 genes; Figure S3 Pie chart of lipid chemical classes. Figure legend indicates the proportion of each lipid; Figure S4 The diagram of lipid data quality assessment. (a) The time series plot of the first principal component in QC samples during an analytical batch; (b) The lipid intensity RSD distribution in QC samples. Figure S5 OPLS-DA score plots in SAT-VAT (a), SAT-AAT (b), and VAT-AAT (c) comparative groups. R2X, R2Y, and Q2Y were the three parameters describing the quality of the OPLS-DA model, where the first two parameters represented respectively the explanatory ability of X variations and Y variations explained by all extracted components, and the third parameter denoted the predictive ability of the model. Table S1 Statistical analysis of transcriptome sequencing of different adipose tissues; Table S2 The differentially expressed genes identified in SAT-VAT group; Table S3 The differentially expressed genes identified in SAT-AAT group; Table S4 The differentially expressed genes identified in VAT-AAT group; Table S5 The overlaps of differentially expressed genes among SAT-VAT, SAT-AAT, and VAT-AAT groups; Table S6 GO annotation of differentially expressed genes identified in SAT-VAT, SAT-AAT, and VAT-AAT groups; Table S7 Pathway enrichment of differentially expressed genes identified in SAT-VAT, SAT-AAT, and VAT-AAT groups; Table S8 Weighted gene co-expression network analysis; Table S9 The enriched GO terms and KEGG pathways for the genes in significant modules; Table S10 The significant GO terms and KEGG pathways related to fat deposition and their involved genes in the lightcyan module; Table S11 The average FPKM expression levels of candidate genes in different adipose tissues; Table S12 The compositions and contents of fatty acid in different adipose tissues; Table S13 Comparison of lipid contents in different adipose tissues; Table S14 The differentially expressed lipis screened in SAT-VAT, SAT-AAT, and VAT-AAT groups; Table S15 The Spearman correlation coefficients between the FPKM expression of 11 candidate genes and the content of important metabolites.

## References

[1] Mantovani G., Bondioni S., Alberti L., Gilardini L., Invitti C., Corbetta S., Zappa M.A., Ferrero S., Lania A.G., Bosari S. (2009). Protein kinase A regulatory subunits in human adipose tissue: Decreased R2B expression and activity in adipocytes from obese subjects. Diabetes.

[2] Trayhurn P., Beattie J.H. (2001). Physiological role of adipose tissue: White adipose tissue as an endocrine and secretory organ. Proc. Nutr. Soc..

[3] Zheng Y., Wang S., Yan P. (2018). The meat quality, muscle fiber characteristics and fatty acid profile in Jinjiang and F1 Simmental×Jinjiang yellow cattle. Asian-Australas. J. Anim. Sci..

[4] Desnoyers F., Pascal G., Etienne M., Vodovar N. (1980). Cellularity of adipose tissue in fetal pig. J. Lipid Res..

[5] Faty A., Ferré P., Commans S. (2012). The acute phase protein Serum Amyloid A induces lipolysis and inflammation in human adipocytes through distinct pathways. PLoS ONE.

[6] Perrini S., Laviola L., Cignarelli A., Melchiorre M., De Stefano F., Caccioppoli C., Natalicchio A., Orlando M.R., Garruti G., De Fazio M. (2008). Fat depot-related differences in gene expression, adiponectin secretion, and insulin action and signalling in human adipocytes differentiated in vitro from precursor stromal cells. Diabetologia.

[7] Misra A., Vikram N.K. (2003). Clinical and pathophysiological consequences of abdominal adiposity and abdominal adipose tissue depots. Nutrition.

[8] Fox C.S., Massaro J.M., Hoffmann U., Pou K.M., Maurovich-Horvat P., Liu C.Y., Vasan R.S., Murabito J.M., Meigs J.B., Cupples L.A. (2007). Abdominal visceral and subcutaneous adipose tissue compartments: Association with metabolic risk factors in the Framingham Heart Study. Circulation.

[9] Bonin M.N., Ferraz J.B., Eler J.P., Rezende F.M., Cucco D.C., Carvalho M.E., Silva R.C., Gomes R.C., Oliveira E.C. (2014). Sire effects on carcass and meat quality traits of young Nellore bulls. Genet. Mol. Res. GMR.

[10] Jiang M., Fan W.L., Xing S.Y., Wang J., Li P., Liu R.R., Li Q.H., Zheng M.Q., Cui H.X., Wen J. (2017). Effects of balanced selection for intramuscular fat and abdominal fat percentage and estimates of genetic parameters. Poult. Sci..

[11] Baldwin R.L., Li R.W., Li C.J., Thomson J.M., Bequette B.J. (2012). Characterization of the longissimus lumborum transcriptome response to adding propionate to the diet of growing Angus beef steers. Physiol. Genom..

[12] Cesar A.S., Regitano L.C., Poleti M.D., Andrade S.C., Tizioto P.C., Oliveira P.S., Felício A.M., do Nascimento M.L., Chaves A.S., Lanna D.P. (2016). Differences in the skeletal muscle transcriptome profile associated with extreme values of fatty acids content. BMC Genom..

[13] Taniguchi M., Guan L.L., Zhang B., Dodson M.V., Okine E., Moore S.S. (2008). Gene expression patterns of bovine perimuscular preadipocytes during adipogenesis. Biochem. Biophys. Res. Commun..

[14] Wang Y.H., Byrne K.A., Reverter A., Harper G.S., Taniguchi M., McWilliam S.M., Mannen H., Oyama K., Lehnert S.A. (2005). Transcriptional profiling of skeletal muscle tissue from two breeds of cattle. Mamm. Genome.

[15] Midelfart A. (2009). Metabonomics--a new approach in ophthalmology. Acta Ophthalmol..

[16] Shajahan-Haq A.N., Cheema M.S., Clarke R. (2015). Application of metabolomics in drug resistant breast cancer research. Metabolites.

[17] Kim S., Kim J., Yun E.J., Kim K.H. (2016). Food metabolomics: From farm to human. Curr. Opin. Biotechnol..

[18] Mahdavi V., Ghanati F., Ghassempour A. (2016). Integrated pathway-based and network-based analysis of GC-MS rice metabolomics data under diazinon stress to infer affected biological pathways. Anal. Biochem..

[19] Sumner L.W., Lei Z., Nikolau B.J., Saito K. (2015). Modern plant metabolomics: Advanced natural product gene discoveries, improved technologies, and future prospects. Nat. Prod. Rep..

[20] Luque de Castro M.D., Quiles-Zafra R. (2020). Lipidomics: An omics discipline with a key role in nutrition. Talanta.

[21] May F.J., Baer L.A., Lehnig A.C., So K., Chen E.Y., Gao F., Narain N.R., Gushchina L., Rose A., Doseff A.I. (2017). Lipidomic Adaptations in White and Brown Adipose Tissue in Response to Exercise Demonstrate Molecular Species-Specific Remodeling. Cell Rep..

[22] Chondronikola M., Volpi E., Børsheim E., Porter C., Saraf M.K., Annamalai P., Yfanti C., Chao T., Wong D., Shinoda K. (2016). Brown Adipose Tissue Activation Is Linked to Distinct Systemic Effects on Lipid Metabolism in Humans. Cell Metab..

[23] Ueda S., Iwamoto E., Kato Y., Shinohara M., Shirai Y., Yamanoue M. (2019). Comparative metabolomics of Japanese Black cattle beef and other meats using gas chromatography-mass spectrometry. Biosci. Biotechnol. Biochem..

[24] Hudson N.J., Reverter A., Griffiths W.J., Yutuc E., Wang Y., Jeanes A., McWilliam S., Pethick D.W., Greenwood P.L. (2020). Gene expression identifies metabolic and functional differences between intramuscular and subcutaneous adipocytes in cattle. BMC Genom..

[25] Connolly S., Dona A., Hamblin D., D’Occhio M.J., González L.A. (2020). Changes in the blood metabolome of Wagyu crossbred steers with time in the feedlot and relationships with marbling. Sci. Rep..

[26] Carrillo J.A., He Y., Li Y., Liu J., Erdman R.A., Sonstegard T.S., Song J. (2016). Integrated metabolomic and transcriptome analyses reveal finishing forage affects metabolic pathways related to beef quality and animal welfare. Sci. Rep..

[27] Wang Y., Chen X., Fan W., Zhang X., Zhan S., Zhong T., Guo J., Cao J., Li L., Zhang H. (2021). Integrated application of metabolomics and RNA-seq reveals thermogenic regulation in goat brown adipose tissues. FASEB J..

[28] Lachmann A., Clarke D.J.B., Torre D., Xie Z., Ma’ayan A. (2020). Interoperable RNA-Seq analysis in the cloud. Biochim. Biophys. Acta Gene Regul. Mech..

[29] Liao Y., Smyth G.K., Shi W. (2014). featureCounts: An efficient general purpose program for assigning sequence reads to genomic features. Bioinformatics.

[30] Varet H., Brillet-Guéguen L., Coppée J.Y., Dillies M.A. (2016). SARTools: A DESeq2- and EdgeR-Based R Pipeline for Comprehensive Differential Analysis of RNA-Seq Data. PLoS ONE.

[31] Rao S., Yu T., Cong X., Lai X., Xiang J., Cao J., Liao X., Gou Y., Chao W., Xue H. (2021). Transcriptome, proteome, and metabolome reveal the mechanism of tolerance to selenate toxicity in Cardamine violifolia. J. Hazard. Mater..

[32] Du L., Li K., Chang T., An B., Liang M., Deng T., Cao S., Du Y., Cai W., Gao X. (2022). Integrating genomics and transcriptomics to identify candidate genes for subcutaneous fat deposition in beef cattle. Genomics.

[33] Wu T., Hu E., Xu S., Chen M., Guo P., Dai Z., Feng T., Zhou L., Tang W., Zhan L. (2021). clusterProfiler 4.0: A universal enrichment tool for interpreting omics data. Innovation.

[34] Ren E., Chen X., Yu S., Xu J., Su Y., Zhu W. (2018). Transcriptomic and metabolomic responses induced in the livers of growing pigs by a short-term intravenous infusion of sodium butyrate. Animal.

[35] Li X., Yin M., Gu J., Hou Y., Tian F., Sun F. (2018). Metabolomic Profiling of Plasma Samples from Women with Recurrent Spontaneous Abortion. Med. Sci. Monit..

[36] Chong J., Wishart D.S., Xia J. (2019). Using MetaboAnalyst 4.0 for Comprehensive and Integrative Metabolomics Data Analysis. Curr. Protoc. Bioinform..

[37] Xiao C., Sun T., Yang Z., Xu W., Wang J., Zeng L., Deng J., Yang X. (2021). Transcriptome landscapes of differentially expressed genes related to fat deposits in Nandan-Yao chicken. Funct. Integr. Genom..

[38] Hanuš O., Samková E., Křížová L., Hasoňová L., Kala R. (2018). Role of Fatty Acids in Milk Fat and the Influence of Selected Factors on Their Variability-A Review. Molecules.

[39] Mannen H. (2011). Identification and utilization of genes associated with beef qualities. Anim. Sci. J..

[40] Samaras K., Botelho N.K., Chisholm D.J., Lord R.V. (2010). Subcutaneous and visceral adipose tissue gene expression of serum adipokines that predict type 2 diabetes. Obesity.

[41] Li Y., Jin D., Xie W., Wen L., Chen W., Xu J., Ding J., Ren D. (2018). PPAR-γ and Wnt Regulate the Differentiation of MSCs into Adipocytes and Osteoblasts Respectively. Curr. Stem Cell Res. Ther..

[42] Liu X., Li S., Wang L., Zhang W., Wang Y., Gui L., Zan L., Zhao C. (2021). The Effect of FATP1 on Adipocyte Differentiation in Qinchuan Beef Cattle. Animals.

[43] Zhang W., Bin Y., Zhang J., Cui L., Ma J., Chen C., Ai H., Xiao S., Ren J., Huang L. (2016). Genome-wide association studies for fatty acid metabolic traits in five divergent pig populations. Sci. Rep..

[44] Sheashea M., Xiao J., Farag M.A. (2021). MUFA in metabolic syndrome and associated risk factors: Is MUFA the opposite side of the PUFA coin?. Food Funct..

[45] Tong L. (2005). Acetyl-coenzyme A carboxylase: Crucial metabolic enzyme and attractive target for drug discovery. Cell. Mol. Life Sci..

[46] Munday M.R., Hemingway C.J. (1999). The regulation of acetyl-CoA carboxylase--a potential target for the action of hypolipidemic agents. Adv. Enzyme Regul..

[47] Zhang S., Knight T.J., Reecy J.M., Wheeler T.L., Shackelford S.D., Cundiff L.V., Beitz D.C. (2010). Associations of polymorphisms in the promoter I of bovine acetyl-CoA carboxylase-alpha gene with beef fatty acid composition. Anim. Genet..

[48] Da Costa A.S., Pires V.M., Fontes C.M., Mestre Prates J.A. (2013). Expression of genes controlling fat deposition in two genetically diverse beef cattle breeds fed high or low silage diets. BMC Vet. Res..

[49] Lengi A.J., Corl B.A. (2007). Identification and characterization of a novel bovine stearoyl-CoA desaturase isoform with homology to human SCD5. Lipids.

[50] Lehnert S.A., Reverter A., Byrne K.A., Wang Y., Nattrass G.S., Hudson N.J., Greenwood P.L. (2007). Gene expression studies of developing bovine longissimus muscle from two different beef cattle breeds. BMC Dev. Biol..

[51] Ntambi J.M., Miyazaki M. (2004). Regulation of stearoyl-CoA desaturases and role in metabolism. Prog. Lipid Res..

[52] Liang H., Xu L., Zhao X., Pan K., Yi Z., Bai J., Qi X., Xin J., Li M., Ouyang K. (2020). RNA-Seq analysis reveals the potential molecular mechanisms of daidzein on adipogenesis in subcutaneous adipose tissue of finishing Xianan beef cattle. J. Anim. Physiol. Anim. Nutr..

[53] Yang A., Larsen T.W., Smith S.B., Tume R.K. (1999). Delta9 desaturase activity in bovine subcutaneous adipose tissue of different fatty acid composition. Lipids.

[54] Siebert B.D., Pitchford W.S., Kruk Z.A., Kuchel H., Deland M.P., Bottema C.D. (2003). Differences in delta9 desaturase activity between Jersey- and Limousin-sired cattle. Lipids.

[55] Matsuzaka T. (2021). Role of fatty acid elongase Elovl6 in the regulation of energy metabolism and pathophysiological significance in diabetes. Diabetol. Int..

[56] Sunaga H., Matsui H., Anjo S., Syamsunarno M.R., Koitabashi N., Iso T., Matsuzaka T., Shimano H., Yokoyama T., Kurabayashi M. (2016). Elongation of Long-Chain Fatty Acid Family Member 6 (Elovl6)-Driven Fatty Acid Metabolism Regulates Vascular Smooth Muscle Cell Phenotype Through AMP-Activated Protein Kinase/Krüppel-Like Factor 4 (AMPK/KLF4) Signaling. J. Am. Heart Assoc..

[57] Matsuzaka T., Shimano H., Yahagi N., Yoshikawa T., Amemiya-Kudo M., Hasty A.H., Okazaki H., Tamura Y., Iizuka Y., Ohashi K. (2002). Cloning and characterization of a mammalian fatty acyl-CoA elongase as a lipogenic enzyme regulated by SREBPs. J. Lipid Res..

[58] Junjvlieke Z., Khan R., Mei C., Cheng G., Wang S., Raza S.H.A., Hong J., Wang X., Yang W., Zan L. (2020). Effect of ELOVL6 on the lipid metabolism of bovine adipocytes. Genomics.

[59] Moisá S.J., Shike D.W., Faulkner D.B., Meteer W.T., Keisler D., Loor J.J. (2014). Central Role of the PPARγ Gene Network in Coordinating Beef Cattle Intramuscular Adipogenesis in Response to Weaning Age and Nutrition. Gene Regul. Syst. Biol..

[60] Ji P., Osorio J.S., Drackley J.K., Loor J.J. (2012). Overfeeding a moderate energy diet prepartum does not impair bovine subcutaneous adipose tissue insulin signal transduction and induces marked changes in peripartal gene network expression. J. Dairy Sci..

[61] Junjvlieke Z., Mei C.G., Khan R., Zhang W.Z., Hong J.Y., Wang L., Li S.J., Zan L.S. (2019). Transcriptional regulation of bovine elongation of very long chain fatty acids protein 6 in lipid metabolism and adipocyte proliferation. J. Cell. Biochem..

[62] Salcedo-Tacuma D., Parales-Giron J., Prom C., Chirivi M., Laguna J., Lock A.L., Contreras G.A. (2020). Transcriptomic profiling of adipose tissue inflammation, remodeling, and lipid metabolism in periparturient dairy cows (Bos taurus). BMC Genom..

[63] Xiong L., Pei J., Chu M., Wu X., Kalwar Q., Yan P., Guo X. (2021). Fat Deposition in the Muscle of Female and Male Yak and the Correlation of Yak Meat Quality with Fat. Animals.

[64] Bonnefont J.P., Djouadi F., Prip-Buus C., Gobin S., Munnich A., Bastin J. (2004). Carnitine palmitoyltransferases 1 and 2: Biochemical, molecular and medical aspects. Mol. Aspects Med..

[65] Sharma S., Sun X., Rafikov R., Kumar S., Hou Y., Oishi P.E., Datar S.A., Raff G., Fineman J.R., Black S.M. (2012). PPAR-γ regulates carnitine homeostasis and mitochondrial function in a lamb model of increased pulmonary blood flow. PLoS ONE.

[66] Houten S.M., Denis S., Argmann C.A., Jia Y., Ferdinandusse S., Reddy J.K., Wanders R.J. (2012). Peroxisomal L-bifunctional enzyme (Ehhadh) is essential for the production of medium-chain dicarboxylic acids. J. Lipid Res..

[67] Ranea-Robles P., Violante S., Argmann C., Dodatko T., Bhattacharya D., Chen H., Yu C., Friedman S.L., Puchowicz M., Houten S.M. (2021). Murine deficiency of peroxisomal L-bifunctional protein (EHHADH) causes medium-chain 3-hydroxydicarboxylic aciduria and perturbs hepatic cholesterol homeostasis. Cell. Mol. Life Sci..

[68] Juge-Aubry C.E., Kuenzli S., Sanchez J.C., Hochstrasser D., Meier C.A. (2001). Peroxisomal bifunctional enzyme binds and activates the activation function-1 region of the peroxisome proliferator-activated receptor alpha. Biochem. J..

[69] Zhang S., Xie C. (2017). The role of OXCT1 in the pathogenesis of cancer as a rate-limiting enzyme of ketone body metabolism. Life Sci..

[70] Kumar H., Srikanth K., Park W., Lee S.H., Choi B.H., Kim H., Kim Y.M., Cho E.S., Kim J.H., Lee J.H. (2019). Transcriptome analysis to identify long non coding RNA (lncRNA) and characterize their functional role in back fat tissue of pig. Gene.

[71] Zeng J., Zhou S.W., Zhao J., Jin M.H., Kang D.J., Yang Y.X., Wang X.L., Chen Y.L. (2019). Role of OXCT1 in ovine adipose and preadipocyte differentiation. Biochem. Biophys. Res. Commun..

[72] Badoud F., Lam K.P., DiBattista A., Perreault M., Zulyniak M.A., Cattrysse B., Stephenson S., Britz-McKibbin P., Mutch D.M. (2014). Serum and adipose tissue amino acid homeostasis in the metabolically healthy obese. J. Proteome Res..

[73] Arden K.C., Viars C.S., Fu K., Rozen R. (1995). Localization of short/branched chain acyl-CoA dehydrogenase (ACADSB) to human chromosome 10. Genomics.

[74] Liu X., Zhang W., Wang H., Zhu L., Xu K. (2021). Decreased Expression of ACADSB Predicts Poor Prognosis in Clear Cell Renal Cell Carcinoma. Front. Oncol..

[75] Ping J., Fang X., Zhao Z., Yu X., Yang R. (2018). The effect of short/branched chain acyl-coenzymeA dehydrogenase gene on triglyceride synthesis of bovine mammary epithelial cells. Arch. Anim. Breed..

[76] Jiang P., Iqbal A., Wang M., Li X., Fang X., Yu H., Zhao Z. (2021). Transcriptomic Analysis of Short/Branched-Chain Acyl-Coenzyme a Dehydrogenase Knocked Out bMECs Revealed Its Regulatory Effect on Lipid Metabolism. Front. Vet. Sci..

[77] Beale E.G., Harvey B.J., Forest C. (2007). PCK1 and PCK2 as candidate diabetes and obesity genes. Cell Biochem. Biophys..

[78] Zhang M., Zheng D., Peng Z., Zhu Y., Li R., Wu Q., Li Y., Li H., Xu W., Zhang M. (2021). Identification of Differentially Expressed Genes and Lipid Metabolism Signaling Pathways between Muscle and Fat Tissues in Broiler Chickens. J. Poult. Sci..

[79] Hakimi P., Yang J., Casadesus G., Massillon D., Tolentino-Silva F., Nye C.K., Cabrera M.E., Hagen D.R., Utter C.B., Baghdy Y. (2007). Overexpression of the cytosolic form of phosphoenolpyruvate carboxykinase (GTP) in skeletal muscle repatterns energy metabolism in the mouse. J. Biol. Chem..

[80] Semakova J., Hyroššová P., Méndez-Lucas A., Cutz E., Bermudez J., Burgess S., Alcántara S., Perales J.C. (2017). PEPCK-C reexpression in the liver counters neonatal hypoglycemia in Pck1 (del/del) mice, unmasking role in non-gluconeogenic tissues. J. Physiol. Biochem..

[81] Forest C., Tordjman J., Glorian M., Duplus E., Chauvet G., Quette J., Beale E.G., Antoine B. (2003). Fatty acid recycling in adipocytes: A role for glyceroneogenesis and phosphoenolpyruvate carboxykinase. Biochem. Soc. Trans..

[82] Beale E.G., Forest C., Hammer R.E. (2003). Regulation of cytosolic phosphoenolpyruvate carboxykinase gene expression in adipocytes. Biochimie.

[83] Xing K., Wang K., Ao H., Chen S., Tan Z., Wang Y., Xitong Z., Yang T., Zhang F., Liu Y. (2019). Comparative adipose transcriptome analysis digs out genes related to fat deposition in two pig breeds. Sci. Rep..

[84] Yamamoto T., Furuhashi M., Sugaya T., Oikawa T., Matsumoto M., Funahashi Y., Matsukawa Y., Gotoh M., Miura T. (2016). Transcriptome and Metabolome Analyses in Exogenous FABP4- and FABP5-Treated Adipose-Derived Stem Cells. PLoS ONE.

[85] Xu D., Shen L., Zhou L., Sha W., Yang J., Lu G. (2020). Upregulation of FABP7 inhibits acute kidney injury-induced TCMK-1 cell apoptosis via activating the PPAR gamma signalling pathway. Mol. Omics.

[86] Bartoň L., Bureš D., Kott T., Řehák D. (2016). Associations of polymorphisms in bovine DGAT1, FABP4, FASN, and PPARGC1A genes with intramuscular fat content and the fatty acid composition of muscle and subcutaneous fat in Fleckvieh bulls. Meat Sci..

[87] Hoashi S., Hinenoya T., Tanaka A., Ohsaki H., Sasazaki S., Taniguchi M., Oyama K., Mukai F., Mannen H. (2008). Association between fatty acid compositions and genotypes of FABP4 and LXR-alpha in Japanese black cattle. BMC Genet..

[88] Shan T.Z., Ren Y., Wu T., Liu C.X., Wang Y.Z. (2009). Regulatory role of Sirt1 on the gene expression of fatty acid-binding protein 3 in cultured porcine adipocytes. J. Cell. Biochem..

[89] Wen S., He Y., Wang L., Zhang J., Quan C., Niu Y., Huang H. (2020). Aberrant activation of super enhancer and choline metabolism drive antiandrogen therapy resistance in prostate cancer. Oncogene.

[90] Ko M., Hattori T., Abdullah M., Gong J.S., Yamane T., Michikawa M. (2016). Phosphatidylcholine protects neurons from toxic effects of amyloid β-protein in culture. Brain Res..

