# Supplementary material for: Transcriptomics and Lipid Metabolomics Analysis of Subcutaneous, Visceral, and Abdominal Adipose Tissues of Beef Cattle
Source: Genes (Basel). 2022 Dec 22;14(1):37. doi: 10.3390/genes14010037 (PMC9858949; doi:10.3390/genes14010037)
Supplement: Supplementary file 1 [file genes-14-00037-s001.zip › genes-2017330-supplementary.pdf]

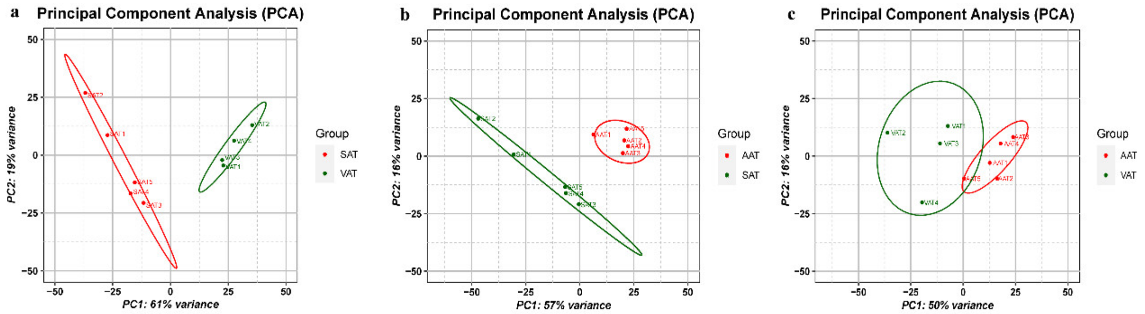

Figure S1. Principal component analyses of samples in SAT-VAT (**a**), SAT-AAT (**b**), and VAT-AAT (**c**) comparative groups.

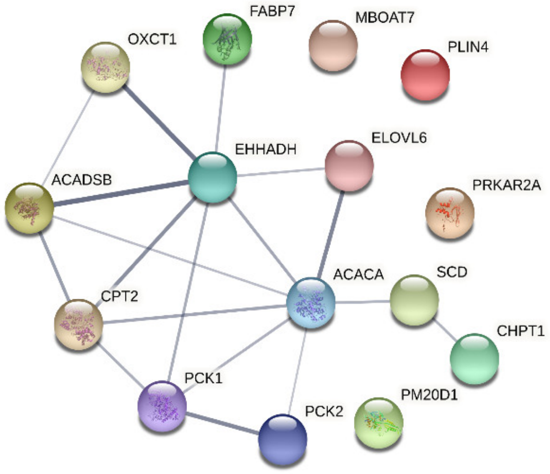

Figure S2. Protein-protein interaction network of 15 genes.

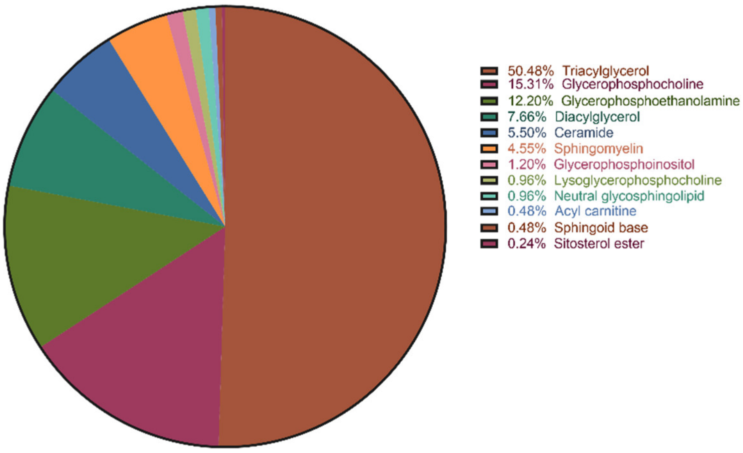

Figure S3. Pie chart of lipid chemical classes. Figure legend indicates the proportion of each lipid.

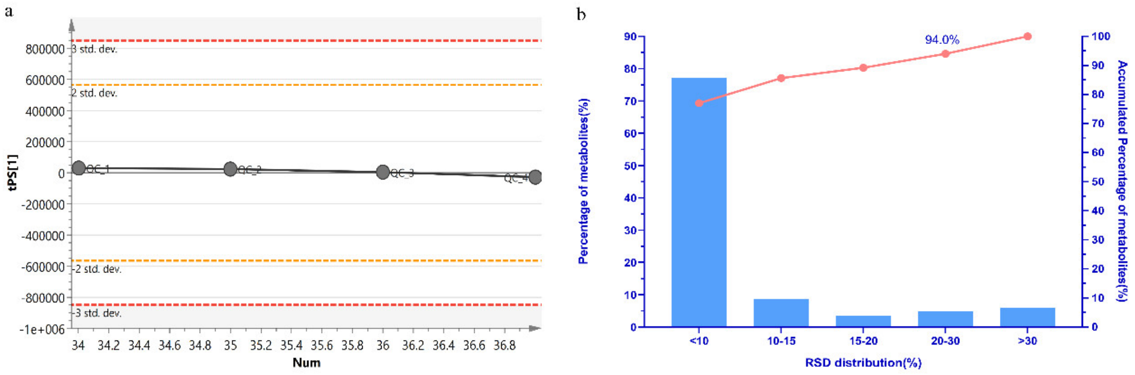

Figure S4. The diagram of lipid data quality assessment. (**a**) The time series plot of the first principal component in QC samples during an analytical batch; (**b**) The lipid intensity RSD distribution in QC samples.

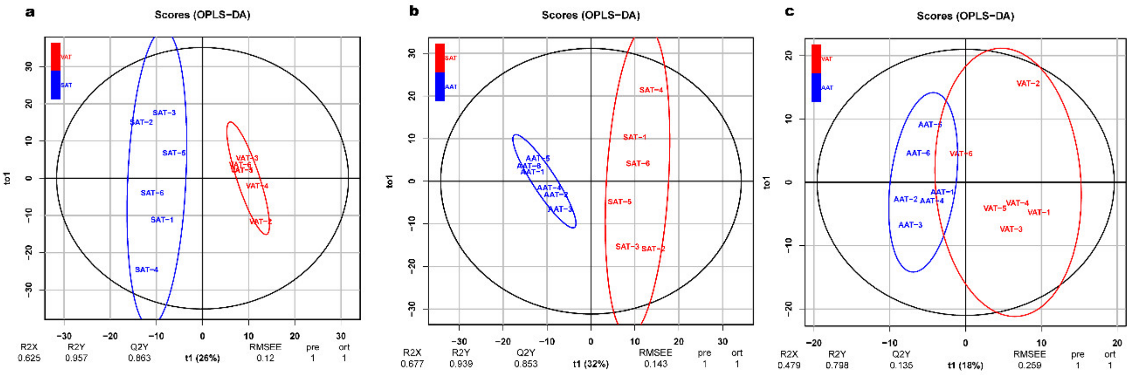

Figure S5. OPLS-DA score plots in SAT-VAT (**a**), SAT-AAT (**b**), and VAT-AAT (**c**) comparative groups. R2X, R2Y, and Q2Y were the three parameters describing the quality of the OPLS-DA model, where the first two parameters represented respectively the explanatory ability of X variations and Y variations explained by all extracted components, and the third parameter denoted the predictive ability of the model.
