# Supplementary figures and images for: Transcriptomics and Lipid Metabolomics Analysis of Subcutaneous, Visceral, and Abdominal Adipose Tissues of Beef Cattle
Source: Genes (Basel). 2022 Dec 22;14(1):37. doi: 10.3390/genes14010037 (PMC9858949; doi:10.3390/genes14010037)

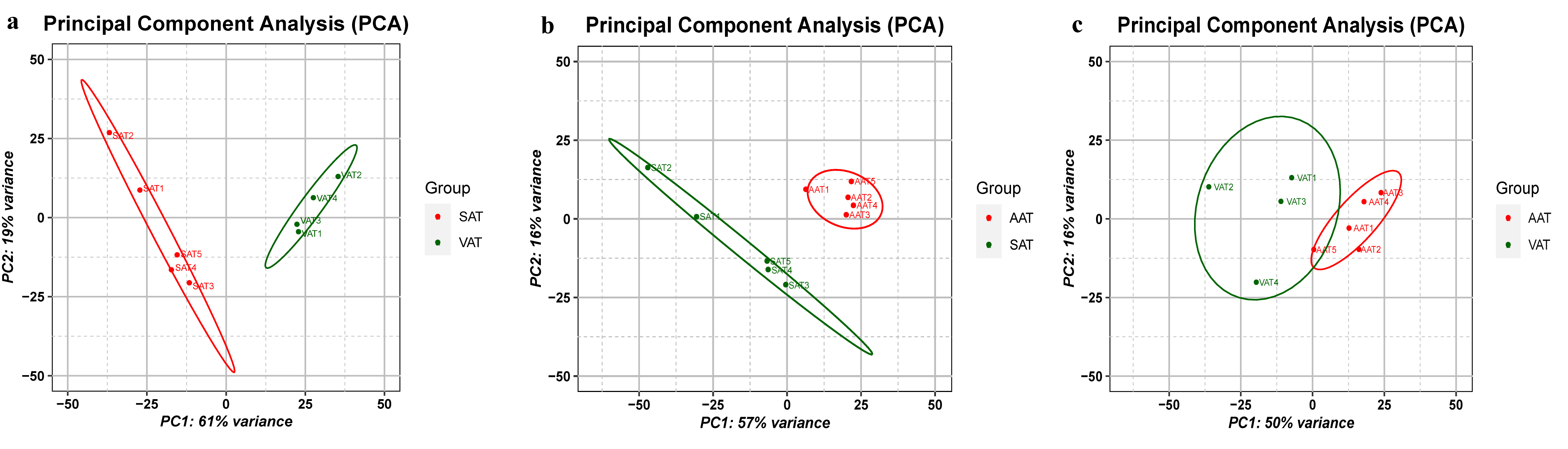

Supplement: Supplementary file 1 [file genes-14-00037-s001.zip › Figure S1.tif]

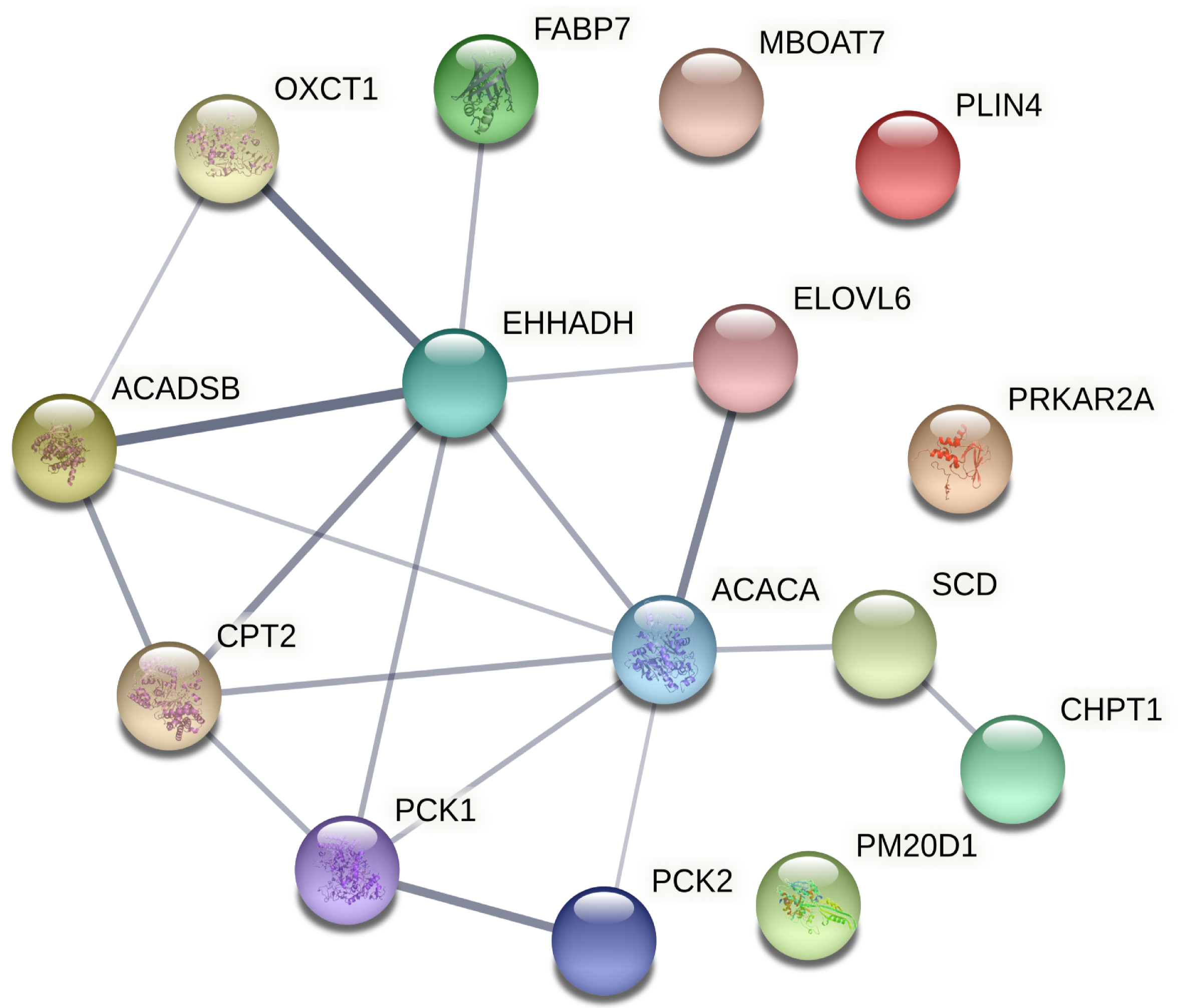

Supplement: Supplementary file 1 [file genes-14-00037-s001.zip › Figure S2.tif]

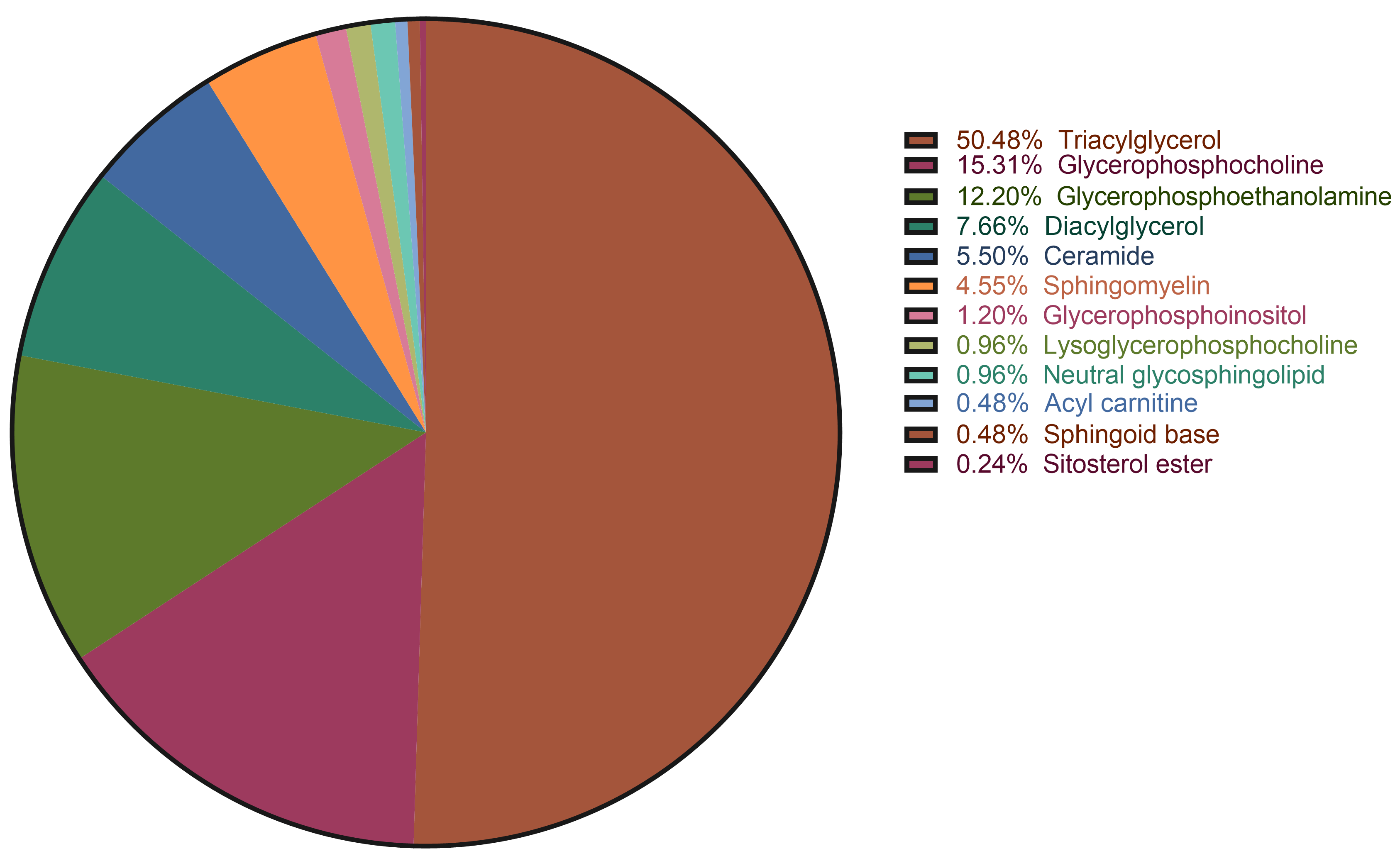

Supplement: Supplementary file 1 [file genes-14-00037-s001.zip › Figure S3.tif]

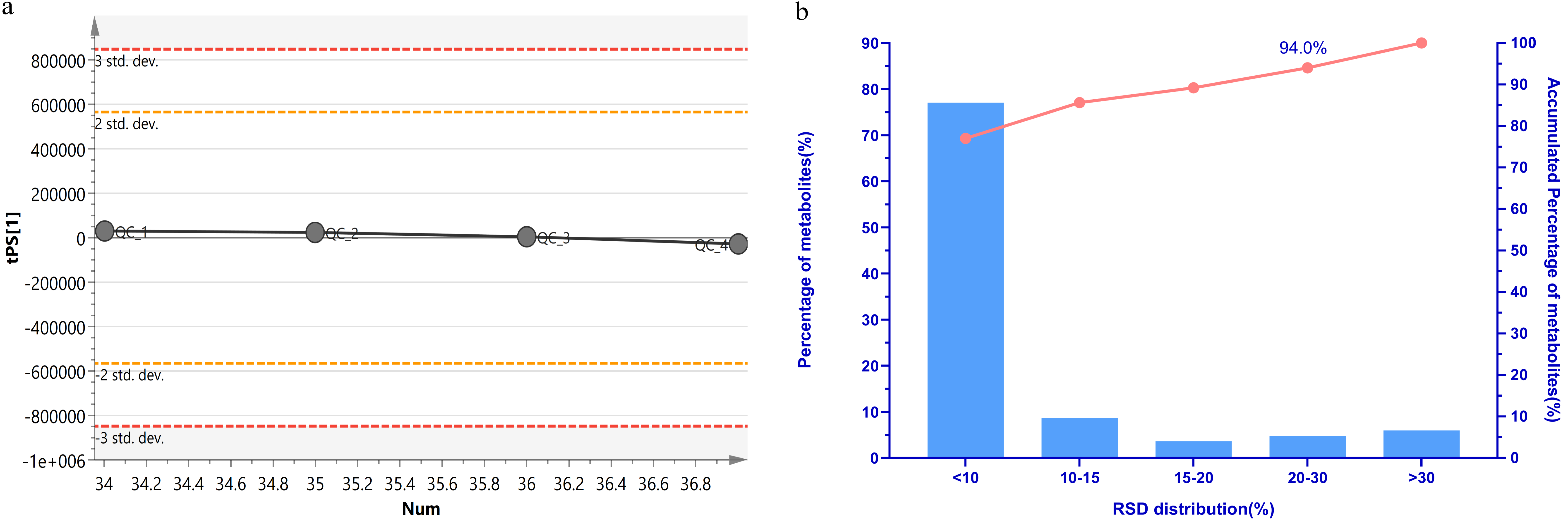

Supplement: Supplementary file 1 [file genes-14-00037-s001.zip › Figure S4.tif]

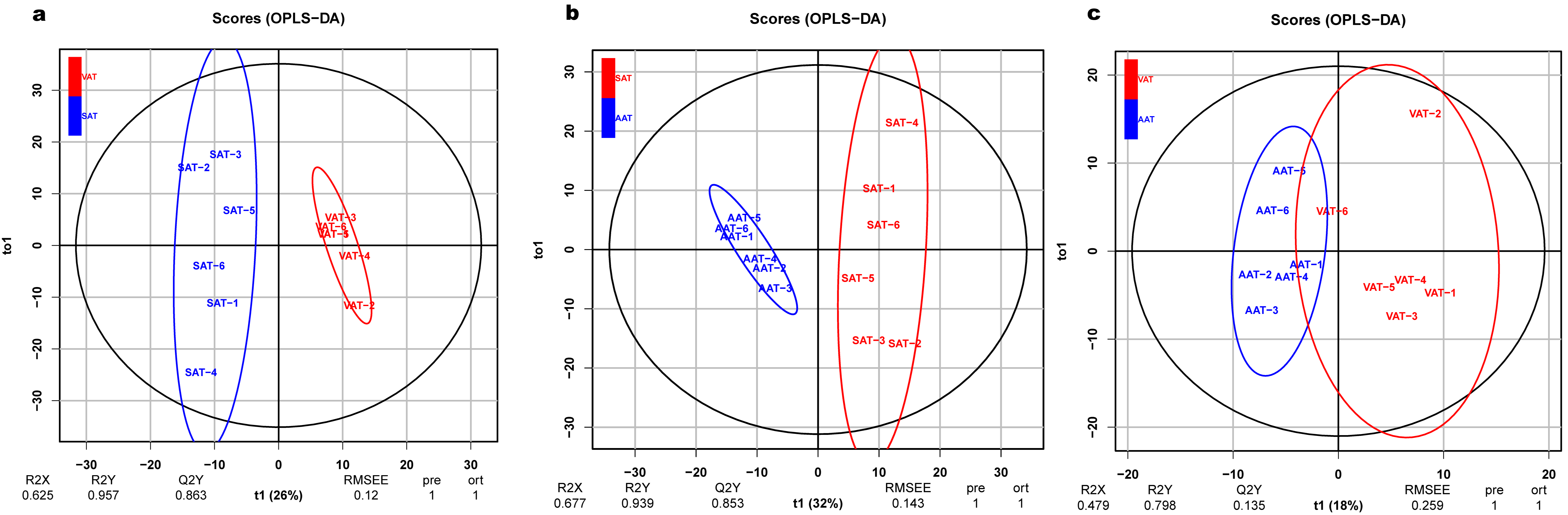

Supplement: Supplementary file 1 [file genes-14-00037-s001.zip › Figure S5.tif]
